# The Crisis of Reproducibility, the Denominator Problem and the Scientific Role of Multi-scale Modeling

**DOI:** 10.1007/s11538-018-0497-0

**Published:** 2018-09-07

**Authors:** Gary An

**Affiliations:** Department of Surgery, University of Chicago, 5841 South Maryland Ave, MC 5091, Chicago, IL 60637 USA

**Keywords:** Crisis of Reproducibility, Multi-scale models

## Abstract

The “Crisis of Reproducibility” has received considerable attention both within the scientific community and without. While factors associated with scientific culture and practical practice are most often invoked, I propose that the Crisis of Reproducibility is ultimately a failure of generalization with a fundamental scientific basis in the methods used for biomedical research. The Denominator Problem describes how limitations intrinsic to the two primary approaches of biomedical research, clinical studies and preclinical experimental biology, lead to an inability to effectively characterize the full extent of biological heterogeneity, which compromises the task of generalizing acquired knowledge. Drawing on the example of the unifying role of theory in the physical sciences, I propose that multi-scale mathematical and dynamic computational models, when mapped to the modular structure of biological systems, can serve a unifying role as formal representations of what is conserved and similar from one biological context to another. This ability to explicitly describe the generation of heterogeneity from similarity addresses the Denominator Problem and provides a scientific response to the Crisis of Reproducibility.

The Crisis of Reproducibility (or Replication Crisis, or a host of similar terms) has been used to describe the phenomenon that a series of foundational studies across a range of disciplines have failed in what can be considered a fundamental aspect of science: that they can be reproduced (Ioannidis 2005; Baker 2016; Prinz et al. 2011; Kaiser 2017; Freedman et al. 2015). The Crisis of Reproducibility has been the subject of many editorials with a series of proposed explanations (Ioannidis 2005; Drucker 2016; Peng 2015; Frye et al. 2015; Jarvis and Williams 2016; Bailoo et al. 2014), with some even questions whether it is a “real crisis” (Fanelli 2018). The vast majority of these opinions fall into two categories:

1. The Crisis of Reproducibility is a by-product of a faulty incentive structure in the current science/academic environment that overly rewards positive results, prompts overstated claims and propagates an “advocacy” mindset that is in opposition to the fundamental role of skepticism in science. The reductio ad absurdum of this position is scientific fraud, which most believe is not widespread; there is a recognition that faulty incentive have a significant role even absent that extreme.
2. The Crisis of Reproducibility is a by-product of sloppy or un-rigorous science, both technically and intellectually, that is further incentivized by #1. Solutions from this branch of opinions gravitate toward the establishment of more rigorous standards for transparency in reporting and publishing methods and results.

While the “societal” factors may certainly play a role in the Crisis of Reproducibility, I have an alternative hypothesis: that the Crisis of Reproducibility is a fundamental epiphenomenon of how biological “science” is carried out, specifically related to a lack of recognition regarding how existing methods do not account for fundamental properties of biological systems. Therefore, I claim that there is a scientific reason for the Crisis of Reproducibility intrinsic to the current process of biomedical research, and that multi-scale mathematical and dynamic computational modeling provides a means of overcoming this deficit.

I assert that the Crisis of Reproducibility is just one of a series of epistemic challenges facing biomedical research over the last several decades; I contend that these are all based on the inability to formally and reliably determine what knowledge can be translated from one context to another. Notable examples of such issues are:

1. The Translational Dilemma or “Valley of Death” in drug development (Butler 2008), which refers to the inability to reliably translate knowledge obtained at the preclinical experimental level into clinically effective therapeutics.
2. Personalized or Precision Medicine (Collins and Varmus 2015), which purports to account for individual–individual variability in the clinical setting by attempting to identify particular patient-disease characteristics associated with improved responsiveness to existing drugs or other treatment modalities. This process is most notably applied in the area of oncology, where tumor genotypes are correlated with presumptively more effective drug combinations [though it is apparent that, based on existing efficacy and applicability, this approach is still in evolution (Marquart et al. 2018)].

I propose that all these issues arise from the same source: a lack of recognition of the Denominator Problem in biomedical research. *I define the Denominator Problem as to the inability to effectively characterize the* “*denominator*” *of a biosystem being studied, where the* “*denominator*” *is defined as the population distribution of the total possible behavior/state space, as described by whatever metrics chosen, of that biosystem.* The concept of a system’s denominator is critical since it is directly tied to the process of generalization of knowledge, a fundamental goal of Science that aims to formally express what is similar and conserved from one observational context to another. The Denominator Problem arises when attempting to answer the question: “When is what I learn from a subset of all possible outcomes of a system generalizable to the system as a whole?” The process of generalization involves dealing with the Problem of Induction: the ability to make reliable statements about a particular phenomenon based on some sampling of that phenomenon.

Science has evolved means of dealing with the Problem of Induction (and, thus, the Denominator Problem, in other fields). One solution to the Problem of Induction is seen in the physical sciences, where “natural laws” have been discovered and characterized in mathematical form; this is a *theory*-*based* approach to science. The other means of addressing the Problem of Induction is through the development and use of statistics. Statistics has evolved as an empirically based means of determining the reliability of generalizing statements, but its application requires knowing the relationship between the empirical sampling and the range of possible phenomena being evaluated, e.g., the denominator set reflecting the space of intended generalization. Therefore, in the absence of theory, achieving the generalizing aim of Science depends upon the reliability of the denominator space of the phenomenon being examined. The use of statistical tools invariably requires an initial assumption about the nature of a system’s underlying total population distribution of observables. When such assumptions have a high degree of confidence, as in the assumption of a normal distribution when the conditions of the Central Limit Theorem are met, characterizing the denominator is not a problem, and traditional statistical tools are very effective. The Denominator Problem, however, occurs when those assumptions cannot be made. I assert that the Denominator Problem in biomedicine arises from a lack of recognizing the full consequences of biological heterogeneity as manifestation of dynamic multi-scale biological processes. The generation of multi-scale biological heterogeneity leads to a vast representational gulf between the empirically collected world of observables (data) and the inferences made regarding the generative processes and structures that produce those observations; this gulf reflects an issue of sparsity arising from limitations in how biological data is produced/collected. A schematic of this problem is depicted in Fig. 1, which shows the relationship between the set of biological possibility (e.g., the denominator of the system), ***A***, versus both an inferred structure of that space based on either empirical sampling ***B*** [i.e., clinical data sets versus the range of possible physiological states, for an example within a research domain see (Button et al. 2013)], and the range of particular possibilities investigated by experiment, ***C1*** and ***C2*** (see Refs. Richter 2017; Richter et al. 2009).

**Fig. 1:**
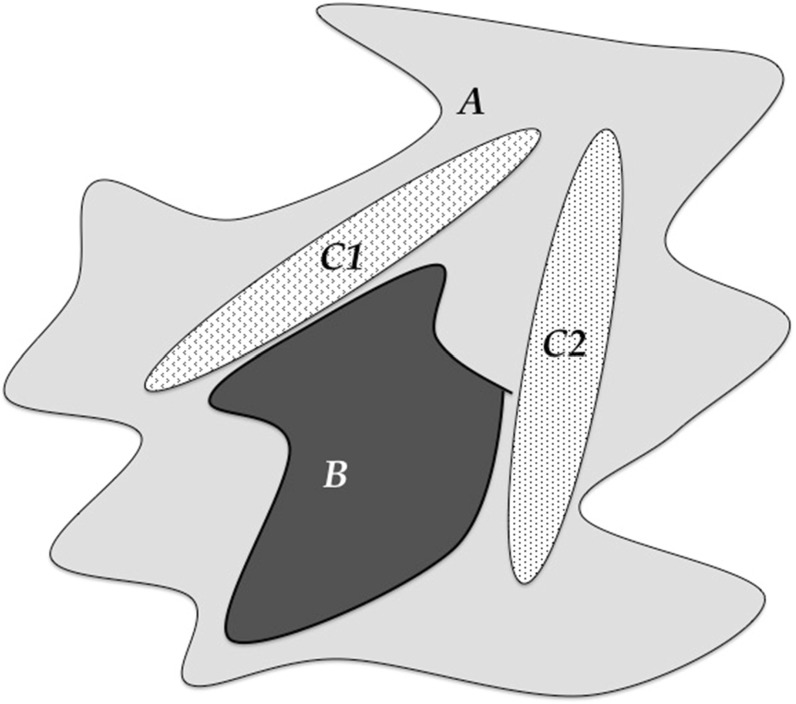
Depiction of the Denominator Problem: the relationship between possible behaviors of a biological system (***A***) with a smaller space of empirical sampling (***B***) and smaller sets examined by “good” experiments (***C1*** and ***C2***). In addition to their lack of coverage, ***B***, ***C1*** and ***C2*** do not reproduce the shape of ***A***. Their inability to characterize ***A*** is the Denominator Problem

The limitations of biological investigation have both a legacy and logistical component [for extensive discussion see Ref. (Vodovotz and An 2014)]. Historically, biology has been an almost completely empirical endeavor, with an emphasis on description at greater and greater levels of detail. Zoology and botany both manifest this property, where similarity and difference are categorized by increasingly subtle descriptions of various features or components of an organism. There is a belief that increasingly detailed information about a particular system equates to increased knowledge of the system, i.e., a quest for *microstate* characterization. The emphasis on various—omics technologies to classify biological states or entities today is a direct result of this paradigm. However, microstate characterization, when used to determine a baseline probability distribution of the denominator of r thusly described (e.g., microstate-based) phenotype space cannot be assumed to have a normal distribution since the variables across the system are no independent and thus violate the conditions of the Central Limit Theorem. Note that this assertion only related to the denominator distribution if viewed as a list of microstate variables, as in the case in various—omics profiles or attempts at biomarker panel discovery. This is not the case for the population of higher-order phenotypes/observables that incorporate and manifest the multi-scale transition from microstate to macrostate (i.e., the normal distribution of human height, for instance). However, with the desire to increase the granularity of state description to the—omics level, there is a loss in the ability to infer the shape of the denominator distribution for the reason described above. Defining the shape of the denominator space through real-world population sampling is further compromised by the sheer logistical challenges of acquiring “good” empirical data: the need for quality control of the collected data produces a paradox in that with better precision of characterization of the data (e.g., “high quality”) the smaller that data set becomes, further degrading the general representative capability of that data; as such Region ***B*** in Fig. 1 shrinks further compared to ***A*** (Button et al. 2013).

At this point it is critical to recognize the importance and role of biology’s foundational theory, *evolution*, its effect on the structure it produces, and the ability to characterize them. Biology is inherently multi-scale, with organizational levels that encompass highly diverse yet somewhat redundant processes. The leads to the case where there is virtually no path-uniqueness between microstates and higher-order phenotypes/observable; in fact, evolution could not work otherwise. This “bowtie” architecture (Csete and Doyle 2004; Doyle and Csete 2011) produces a modular organization for biology that allows the persistence of a diversity of potential in the face of natural selection optimizing on phenotypic fitness. The lack of path-uniqueness in how generative microstates produce phenotypic macrostates means that traditional reductionist methods of system characterization insufficiently capture the true diversity (and therefore the effective denominator space in terms of microstate) of a biological system: what is identified through classical reductionism is only a sliver of plausible solutions/outcomes. This dynamic is reflected in Fig. 1, where ***C1*** and ***C2*** are intentionally draw narrower than ***A*** or ***B*** to illustrate an intrinsic inability of reductionist experimental biology to effectively reflect system denominator space. There are inherent constraints imposed by what constitutes a “good experiment” on the ability of those experiments to approach representing A, namely the need to produce highly controlled conditions that limit variability in order to strengthen the statistical power of experimental results. In other words, these studies explicitly limit the range of possible phenotypes represented with the experimental system for the sake of a more definitive conclusion arrived at through the specific experimental preparation. While this paradigm is essential for experimental biology, but its very nature it produces the preconditions of the Crisis of Reproducibility: experimental results may be statistically valid with respect to the very specific context in which they were generated, but this increased precision comes at the expense of increased sensitivity to subtly different conditions that may lead to disparate results (Richter 2017). A practical manifestation of this phenomenon is seen in the extreme sensitivity to initial conditions in experimental biology, and is illustrated by the following situation familiar to anyone who has run a basic science research laboratory: a previously reliable and reproducible experimental model needs to be nearly completely recalibrated when either a new laboratory tech/post-doc/grad-student starts, or when the bedding/reagent supplier changes, or even the air or water filters for the laboratory are changed. In fact, this sensitivity to initial/experimental conditions is exactly how the Crisis of Reproducibility came to be recognized, as meticulously followed experimental procedures intended to replicate prior results were unable to do so (Begley and Ellis 2012). The limitations of experimental biology are further accentuated by the fact that the knowledge extracted from these experiments is focused on component-based description, e.g., state identification. Such an approach necessarily results in systems that are viewed, at best, as a series of static snapshots of their component configuration (i.e., metabolic state, gene/mRNA/protein expression levels, receptor levels/types, histological features, biomarker panels, etc.), but without any explicitly described process linking these various snapshots.

However, biological systems are not static; they are dynamical systems. Biological phenomena consist of trajectories that define the progression of one state to another. Thus, the dynamics of biology requires its characterization through functions. I assert that these functions, operating both within and across scales of organization, are what are conserved in biology. This conservation results in the path-non-uniqueness and modular redundancy seen in biology’s bow-tie organizational structure that allows evolution to work. This function-based view of similarity exactly reflects the role of theory in the physical sciences, where natural laws in physics and chemistry have mathematical forms able to generate the vast range of heterogeneous instances seen in the physical world. It is worth noting that there are antecedents for thinking about biology in terms of functions, namely in classical physiology and classical genetics. They both view biology in terms of generalizable functions amenable to abstraction (where it is recognized that abstraction = generalization). But with the advent of molecular biology, these function-based representations of biology have become subsumed by the desire for increasing descriptive detail, to a point where the representational capabilities of classical physics and genetics have broken down. The fact is that, at the level of granularity biological systems are currently being studied, the dynamics and heterogeneity of their behavior has proven too complex to be characterized by unifying natural laws using existing mathematical methods (at least thus far). As such, modern biology (especially biomedicine) has retreated to its historical descriptive legacy, focusing more on the differences between systems as opposed to trying to discover more powerful generalizing perspectives. I propose that the solution to this divergence from a function-based view of biology is to employ mathematical and dynamic computational models that balance descriptive detail with generalizing abstraction to represent and instantiate conserved functions. When used to represent complex biological objects by mapping in a modular fashion to the multiple levels of organization seen in those objects, multi-scale models (MSMs) are able to encapsulate what is conserved from one biological instance to another. Consider the diagram depicted in Fig. 2. The upper row of ovals represent that traditional reductionist investigatory workflow, where laboratory experiments are carried out in a successive series of biological model systems of increasing complexity and fidelity to the clinical target. However, the mapping/transfer of knowledge from one context to the other is an incomplete injective relationship at best, i.e., there is an inferred partial mapping from the components/processes from one model to another, but it is both incomplete (non-comprehensive) and loosely specified. This is because the biological models are opaque, and consist of numerous “hidden” processes not accounted for in the mapping from both the domain object and the codomain object; this uncertainty is represented by the “?” over each injective arrow. Alternatively, the in silico representations (i.e., models) of the knowledge assumed at each level are transparent: there are no “hidden” variables. Also, while in silico model is necessarily a reduced incomplete representation of the real-world system, its explicit and transparent composition makes it a proper subset (PS). As such, the injective relationship between the in silico object (domain) and its target real-world object (codomain) is an explicit injection; the unrepresented aspects of the real-world object are “ignored” for the purposes of the mapping as long as selected macrostate phenotypes are generated of sufficient fidelity by the in silico analog (*For obvious reasons, the mapping cannot occur at the microstate-level, since it is acknowledged that the in silico object does not contain every feature of the real-world object). Furthermore, since the in silico objects are completely transparent and explicitly specified, the relationship along the lower horizontal axis is an explicit bijective relationship. There is a caveat that increasing complexity of the in silico models may require incorporation of new features not present at the lower level model, and therefore, result in a partial bijective relationship from one specific in silico model to the more complex one, but this process should take advantage of the modular nature of biology, allowing the integration of subset in silico objects such that in combination/union they form an explicit bijective relationship with the more complex in silico objects. This allows the nesting of in silico objects that move toward those representing clinical populations. The key here is that the entire sets of the domain and codomain are explicitly specified and formally expressed, which significantly strengthens any statements made about their behavior. This is consistent with how mathematical formalisms are used in the physical sciences, where target systems are explicitly specified as being governed by one or more natural laws. Used in an analogous fashion, MSMs hold the key to addressing the Denominator Problem and thus the Crisis of Reproducibility. MSMs are able to generate multiple instances of a system given an explicitly specified function, producing data at a scale not feasible in real-world systems. This scale of data generation addresses the limitations of Letter ***B***, where there is virtually no limit to the sample size acquired, while encompassing and expanding on the representational capacity of Letters ***C1*** and ***C2***, to produce a denser and more expansive baseline population distribution (e.g., the denominator space) of the system being studied (see Fig. 3). The distribution of the denominator space is defined by the sum of the trajectories generated from the parameter space of the model, which incorporates stochasticity (both epistemic and aleatory). Thus, observed biological heterogeneity is a manifestation of the parameter space of an underlying MSM, and advances in computational power allow the scale of simulation experiments needed to more fully characterize the denominator of any biological system being studied.

**Fig. 2:**
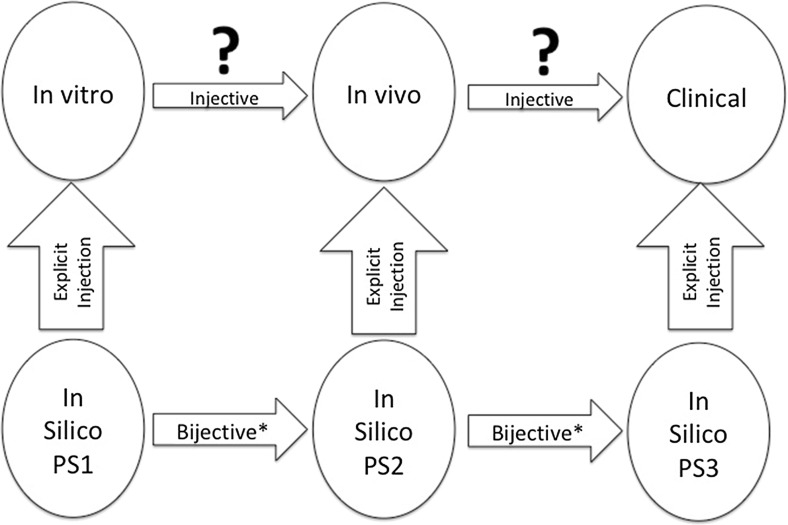
Role of MSMs in determining what is similar across biological instances. Since biological objects are opaque, the injective mapping across them is uncertain (upper row). However, mappings between in silico analogs/modules are explicit injections, and since they are transparent (Proper Subsets = PS) the mapping across modules is explicit and bijective (or *partially bijective)

**Fig. 3:**
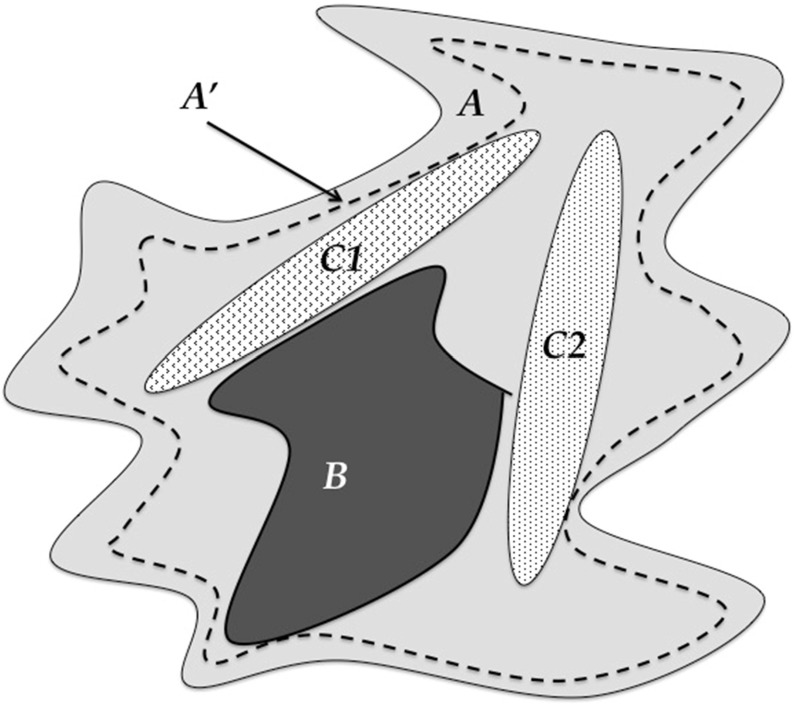
Depiction of the ability of MSMs to more completely address the Denominator Problem. ***A′*** (the region enclosed by the dashed line) represents the potential unifying descriptive capacity offered by computational MSMs serving as surrogates for the real system. Note that ***A′*** remains an approximation of ***A***, that will improve with iterative refinement over time (***A′ *→ *A***)

As an example, we have applied these concepts in a preliminary fashion to the problem of sepsis (Cockrell and An 2017). We used a previously validated agent-based model (ABM) that simulates the innate immune response as a proxy system to characterize the denominator space of that system’s response dynamics to infectious insults. We utilized high-performance computing to perform extensive parameter space characterization of the ABM by simulating over 66 million simulated patient trajectories, each trajectory made up of up to 216,000 time points representing 90 days of simulated time, and in so doing were able to define a region of parameter space consistent with plausible biological behavior, essentially creating a 1st approximation of the “true” denominator space of sepsis. As a comparison, there have been roughly 30,000 patients enrolled in all the reported clinical trials for sepsis (Buchman et al. 2016), data that exists in general at a few time points and a limited set of observables. Furthermore, the generated state space for sepsis demonstrated a complex, multi-dimensional topology requiring the development of a novel metric for characterization [Probabilistic Basins of Attraction or PBoA (Cockrell and An 2017)], and definitely not consisting of a normal distribution, or one that could have reasonably been inferred a priori. Further analysis of this near comprehensive behavior space characterization demonstrated that any attempt to develop a state-based sampling strategy that would be predictive of system outcome, the motivation behind biomarker discovery, was futile (Cockrell and An 2017). This suggests that attempts for reliable forecasting sepsis trajectories would have to adopt some means of model-based, non-linear classifier. Ongoing work in this area is aimed at utilizing advanced learning and optimization techniques to discover both forecasting classifiers and multi-modal control strategies (Cockrell and An 2018; Petersen et al. 2018).

The following statements summarize the reasoning provided in this paper as to how the Crisis of Reproducibility arises out of the Denominator Problem inherent to how biomedical research is currently conducted:

1. The shape of the denominator space for a microstate characterization for a complex biological system cannot be assumed.
2. Insufficient sampling of that denominator space will lead to an inability to generalize knowledge generated from that sampling, i.e., an inability to either translate knowledge from one context to another, or reproduce one experiment in another context.
3. The paradigm/requirements of experimental biology serve to constrain the sampling of denominator space. This same paradigm leads to extreme sensitivity to conditions and irreproducibility.
4. The logistical barriers of clinical research limit the density/granularity of representation and characterization denominator space (e.g., sparsity).
5. Multi-scale models can and need to be used as proxy systems that can both bind together experimental knowledge (e.g., link denominator spaces generated by reductionist experiments to define the shape of the overall space) and “fill in the gaps” of clinical data (e.g., increase the density coverage of the denominator space).

There is, however, a significant cautionary note with respect to the use of MSMs for this unifying purpose. Specifically, an emphasis on precise and detailed prediction as a means of judging the adequacy and validity of MSMs generates the same limitations that afflict experimental biology: trying to enhance precision by reducing or eliminating output variability or noise functions to restrict the denominator space represented by the MSM and reduces its generalizing capability. This phenomenon most often manifests with over-fit and tightly parameterized, brittle models; therefore, overcoming this trap requires employing the concept of using widely bounded parameter spaces as part of the description of a MSM, and utilizing experimental/clinical data that incorporate outliers that reflect a sparse sampling of the wider behavioral denominator space.

In this fashion, MSMs serve a critical fundamental role in the scientific process: they are able to generate the data seen in different contexts (be it in different experiments or clinical situations) using the same functional structure through either expansion of parameter space or stochastic processes. As such, these models function as (t)heories that encapsulate what can be considered “similar” between ostensibly different biological systems or individuals, thereby obviating the “crisis” of obtaining robust, scalable and generalizable knowledge in biomedical research. With a wider adoption of this approach to using MSMs, it is hoped that perhaps new, more powerful mathematical formalisms will be identified that are able to more effectively depict biological systems in all their richness. In the meantime, however, I suggest that the use of MSMs as described above can bring a much needed level of formalism to determining what is and is not similar between different biological systems, be it across the translational divide (to address the Valley of Death) or between individuals for “true” Precision Medicine, which should mean the right drugs in the right combinations for the right patients at the right time. Having trustworthy formal functional representations that can effectively capture biological heterogeneity is a necessary step in being able to apply true engineering principles to identifying strategies to control pathophysiological processes back to a state of health.
